# Scientific Mapping of Chia Protein Research: State of the Art and Future Trends

**DOI:** 10.3390/foods13244181

**Published:** 2024-12-23

**Authors:** Júlia Scherer Santos, Arthur Coelho Durso, César Augusto Sodré da Silva, Rejane de Castro Santana

**Affiliations:** 1 Department of Chemistry, Federal University of Viçosa, Viçosa 36570-900, MG, Brazil; julia_scherer_santos@hotmail.com (J.S.S.); arthur.durso@ufv.br (A.C.D.); 2 Department of Food Technology, Federal University of Viçosa, Viçosa 36570-900, MG, Brazil; cesar.sodre@ufv.br

**Keywords:** bibliometric, peptide, techno-functional, functional foods, *Salvia hispanica*

## Abstract

This report is a pioneering bibliometric analysis of chia proteins as well as a review of the current/future themes of chia proteins. Reports were selected from the Scopus database, and networks of co-word occurrence, co-cited references, and the bibliographic coupling of documents were obtained. The health benefits and functional properties of chia proteins/peptides are current themes while the research of chia peptides is an emergent theme. The co-word network showed a low link between health benefits and functional properties, concluding that protein derivatives with both properties must be better explored. This review elucidated how the conditions of protein extraction and protein hydrolysis must be adjusted to maximize the intended functional characteristics and health benefits. An extremely alkaline pH and heating provided chia proteins with the lowest solubility, emulsifying properties, and antioxidant activity. Higher hydrolysis time increases peptides’ hydrolysis degree, which affects its biological activity. Moreover, chia peptides showed higher oil absorption and emulsifying properties than chia protein isolates. The highlighted findings of this report represent the current research status which may require a new updated review in the future. A more in-depth approach to chia protein derivatives’ extraction will enable their quick development in the food, pharmaceutical, and cosmetic industries.

## 1. Introduction

Currently, chia seeds (*Salvia hispanica*) have been studied as a good source of natural antioxidants, protein, unsaturated oil, proteins, and dietary fiber and therefore are a potential ingredient for functional foods [1,2]. Chia proteins may improve food functionalities such as emulsification, foaming, water, and oil-holding capacities [1] as well as promote antioxidants [3,4], anti-inflammatory activity [4], and blood-pressure lowering [5]. In turn, chia peptides have shown promising application in foods due to their ability to scavenge oxygen free radicals, reduce blood pressure, and stimulate insulin secretion [5].

Extraction and hydrolysis conditions play a significant role in determining the characteristics of chia proteins and peptides. Factors such as pH and temperature during extraction impact the composition of amino acids and the protein’s structural configuration. These changes subsequently affect the protein’s techno-functional attributes and potential application in food products [6,7]. Chia proteins are obtained after milling the seeds and removing the mucilage and the oil, obtaining degummed flour and defatted flour, respectively [1]. Chia peptides are obtained mainly from the hydrolysis of chia proteins [8,9].

Hence, a pioneering bibliometric analysis of chia proteins and peptides reports was performed aiming to identify the research status of chia proteins and peptides. Maps of co-word occurrence, co-cited references, and the bibliographic coupling of documents were constructed by using Vosviewer software. Co-citation and bibliographic coupling networks were employed to detect older and current research themes, respectively. Co-word network analysis was used to detect the keywords most described by authors. Furthermore, the analysis of bibliometric maps was used to detect emerging themes and topics that are still little explored. Furthermore, an overview of the main aspects covered by current and future research themes is also provided, including protein extraction conditions, techno-functional properties, and the health-beneficial characteristics of protein and chia peptides, contributing to the future application of chia proteins and derivatives by the food industry.

## 2. Methods

**Figure 1:**
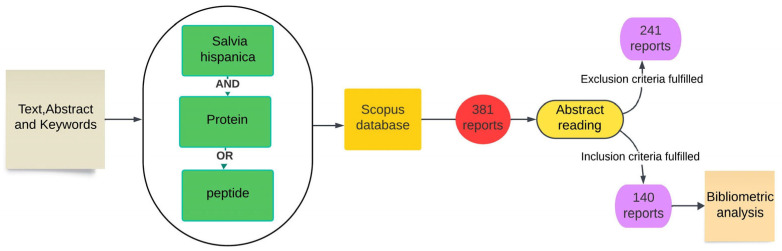
Search methodology and selection of reports for bibliometric analysis.

Reports from the Scopus database were searched using the terms “*Salvia hispanica*” and “protein” or “peptide” in the article text, abstract, and keywords. Documents mentioning any chia protein derivative for food application, in any year of publication, were selected. Reports mentioning other non-food applications or those not involving chia proteins or peptides were excluded from the search. The Scopus database was chosen for its extensive collection of publications across various fields and wide-ranging coverage of the literature. The selected report data were exported from the database and inserted into the Vosviewer^®^ software (1.6.19). After searching for reports in the Scopus database, 381 documents were retrieved, including review articles, book chapters, and conference proceedings. A total of 140 reports were selected after applying inclusion and exclusion criteria. Figure 1 summarizes the search methodology in the Scopus database.

Networks of co-word occurrence, co-citations of cited references, and the bibliographic coupling of documents were obtained. Vosviewer^®^ arranged the data into clusters composed of several nodes. In the co-word network, the nodes are keywords. For co-citation and bibliographic coupling, nodes were selected from the Scopus database. Additionally, the link and total link strength values were obtained, where the link is a numerical value that measures the connection between two data. The total link strength is the sum of all the links.

Network and overlay visualizations of co-word occurrences were obtained from the keywords defined by the authors using a minimum threshold of five concomitant keyword occurrences. Duplicated keywords recovered by the co-word occurrence analysis were eliminated using the thesaurus file. Synonyms were also removed. *Salvia hispanica* L. and *Salvia hispanica* were replaced by chia. Antioxidant activity was replaced by antioxidant. Chia seed and chia seeds were replaced by chia. Proteins were replaced by protein. Bioactive peptides were replaced by peptides. Protein hydrolysates were replaced by peptides. For co-citation and bibliographic coupling networks, a minimum of 5 and 125 citations were selected, respectively. A thesaurus file was employed to remove duplicated references from co-citation analysis and to standardize the presentation of report data in the following format: surname of the first author (year of publication). Overlay and network visualizations of co-word occurrences were employed to determine the current and main keywords. Co-citation identified retrospective research themes and bibliographic coupling analysis identified current and future research themes.

The publications and citations metrics from the last 10 years were also performed. The search of “*Salvia hispanica*” and “protein” was performed between 2014 and 2024 in title, abstract, and keyword aiming to access chia protein metrics. For peptides, the search of “*Salvia hispanica*” and “peptide” was accomplished between 2014 and 2024 also in the title, abstract, and keywords.

## 3. Network Mapping and Publication Metrics of Chia Proteins

**Figure 2:**
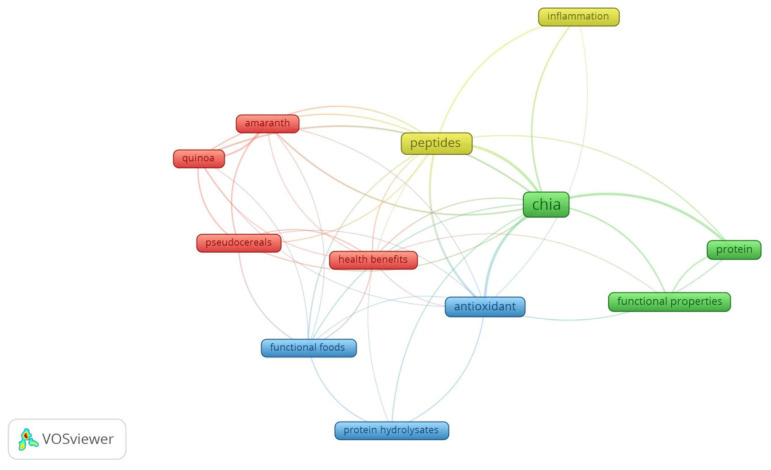
Network visualization of co-word occurrence obtained from Vosviewer software^®^ from 140 reports selected from the search of “*Salvia hispanica*” and “protein” or “peptide” on Scopus database.

Firstly, keyword analysis was used to determine the words most used by authors. The network co-occurrence visualization (Figure 2) shows the presence of four clusters. Cluster 1 includes amaranth, health benefits, pseudocereals, and quinoa. Cluster 2 includes the following keywords: chia, functional properties, and protein. Cluster 3 is composed of antioxidant, functional properties, and protein hydrolysates. Cluster 4 contains the following words: inflammation and peptides.

The most frequent words include chia, peptides, and antioxidant. Chia had a total of 59 co-occurrences, and it was linked with 11 other keywords. Peptide had a total of 26 occurrences while antioxidant had a total of 21 occurrences. Peptide and antioxidant are linked to 10 other words. There is a strong relationship between the use of chia or its derivatives and health properties since the term health benefits is linked to other eight terms, including chia and peptides. Chia and peptides had the highest strength link of 62 and 41, respectively, denoting their higher co-occurrence with other terms.

As chia, other pseudocereals terms were included in cluster 1: amaranth and quinoa. They have been recently investigated as food functional ingredients, promoting health benefits [10] as well as improving functional properties [1,10]. Hence, chia, amaranth, and quinoa are linked to functional foods (Figure 1). Functional foods combine both health benefits and adequate nutritional properties, while functional properties of foods are associated with food processing, aiming to improve the overall food features [10].

Peptides, antioxidant, and chia are concomitant with the following other words: protein hydrolysates, pseudocereals, amaranth, quinoa, protein, and inflammation (Figure 2). Chia seeds are used to obtain proteins and protein hydrolysates. Chia protein hydrolysis originates from peptides [11,12,13]. Due to chia’s antioxidant ability, a reduction in inflammation is noted [14]. As many diseases are related to inflammation and oxidative stress, the antioxidant and anti-inflammatory properties are related to health benefits.

Functional properties were linked only to proteins (with a link strength of 3), but not to peptides. In addition, there was a link strength of 2 between protein and antioxidant (Figure 2). The lower strength value between protein and antioxidant shows that are few investigations regarding the antioxidant activity of chia proteins. The absence of a link between functional properties and peptides evidences that the functional properties of peptides are underexplored.

**Figure 3:**
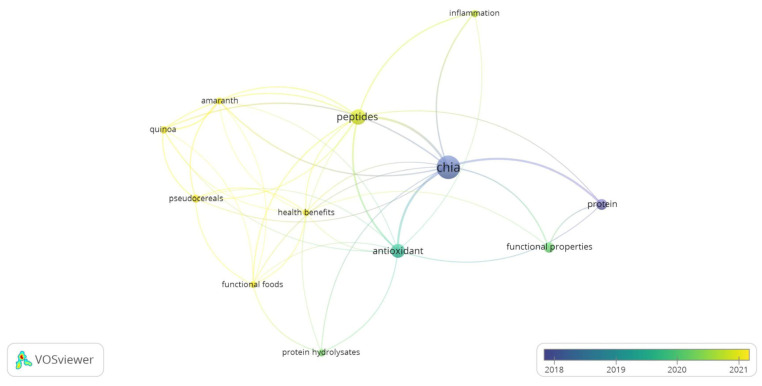
Overlay visualization of co-word occurrence obtained from Vosviewer software^®^ from 140 reports selected from the search of “*Salvia hispanica*” and “protein” or “peptide” on Scopus database.

In addition, as shown in Figure 3, the research on health benefits, functional foods, inflammation, and peptides is more recent than that on chia and protein research. As peptides are produced from proteins or chia flour, research concerning the entire chia seed and the protein component should be previously realized. The recent use of peptides and functional foods keywords (Figure 3) denotes their greater frequency in reports and therefore evidences their research interest and future trending theme. The overlay distribution of the other keywords that are dated between 2018 and 2021 are also current keywords.

**Figure 4:**
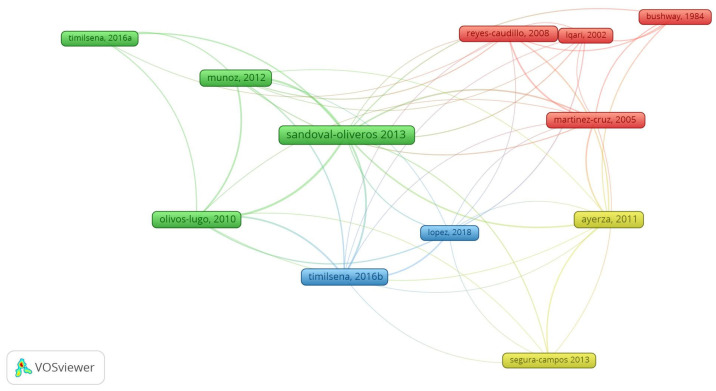
Twelve co-cited references obtained from Vosviewer software^®^ from 140 selected reports on the search of “*Salvia hispanica*” and “protein” or “peptide”.

Networks of co-cited references are shown in Figure 4, for which a total of 12 co-cited documents were arranged into four clusters. The most co-cited report [15] had 26 citations. It is one of the first to demonstrate chia protein isolation, especially globulins and albumins [15], and therefore it is a fundamental one in the research of proteins and peptides from chia. The second most cited reports [16] had a total of 11 citations, and it approaches chia seed features and the extraction of mucilage.

The theme research and the co-cited reports for each cluster of Figure 4 are displayed in Table 1. There is a temporal evolution of the research, starting with the cluster 1 theme (plant bioactive compounds) [17,18,19,20], followed by the themes of cluster 4 (health benefits of chia seed and its byproducts) [11,21], cluster 2 (characterization of chia byproducts) [15,16,22,23], and ultimately cluster 3 (chia protein characteristics and functional properties) [24,25].

**Table 1:** Reports from each cluster of the co-citation network obtained from Vosviewer software.

Cluster Number	Reports	Reference
1	Bushway (1984)	[17]
	Lqari (2002)	[18]
	Martínez-Cruz (2014)	[19]
	Reyes-Caudillo (2008)	[20]
2	Sandoval-Oliveros (2013)	[15]
	Muñoz (2012)	[16]
	Olivos-Lugo (2010)	[22]
	Timilsena (2016a)	[23]
3	Timilsena(2016b)	[24]
	López (2018)	[25]
4	Ayerza (2011)	[21]
	Segura-Campos (2013)	[11]

**Figure 5:**
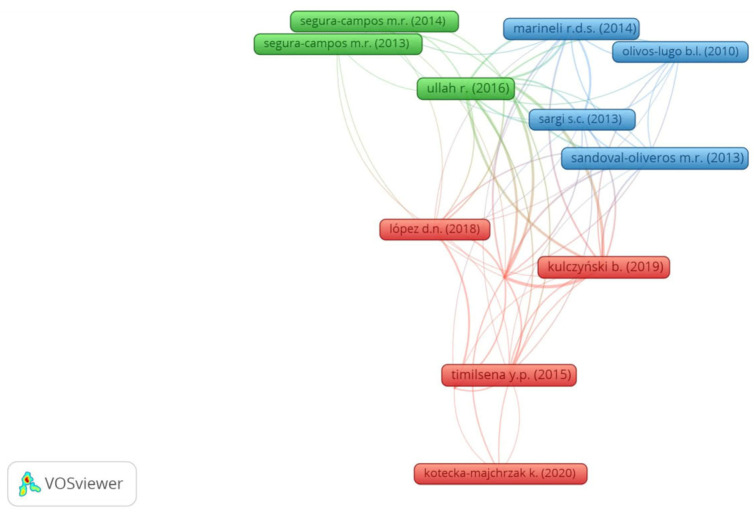
Bibliographic coupling of 14 references obtained from Vosviewer software^®^ from 140 selected reports on the search of “*Salvia hispanica*” and “protein” or “peptide.

Regarding the bibliographic coupling (Figure 5), 14 reports, distributed into three clusters have shared references. Likewise, the co-cited network, themes, and reports from each bibliographic coupling cluster were settled (Table 2). The following themes were addressed: characterization and health benefits of chia seed proteins/peptides (cluster 1), health benefits and functional properties of chia seeds and derivatives (cluster 2), and characterization and chemical composition of chia seeds and derivatives (cluster 3). The most cited reports belong to cluster 3, with 238 citations [26]. This report is important because it addresses the chemical composition of chia seeds [26].

Although the bibliographic coupling network shows the current research themes (Table 2), the average publication date of each cluster shows that the oldest theme is that from cluster 3 while the theme of cluster 1 is the most recent. Chia seed chemical composition (cluster 3) [15,23,26,27] must be previously determined to enable its use or its derivatives’ use in foods [11,23,25,28,29,30,31,32,33]. The theme of chia seed and derivative characterization (cluster 3) is older than the health benefits and functional properties of chia seeds and derivatives (cluster 2).

**Table 2:** Reports from each cluster of bibliographic coupling network obtained from Vosviewer software.

Cluster Number	Reports	Reference
1	Grancieri (2019)	[31]
	Kulczyński (2019)	[28]
	Kotecka- Majchrza (2020)	[32]
	López (2018)	[25]
	Timilsena (2015)	[34]
	Timilsena (2016)	[24]
2	Muñoz (2013)	[33]
	Segura-Campos (2013)	[11]
	Segura-Campos (2014)	[29]
	Ullah (2016)	[30]
3	Marinelli (2014)	[26]
	Olivos- Lugo (2010)	[22]
	Sandoval- Oliveros (2013)	[15]
	Sargi (2013)	[27]

Furthermore, by comparison of co-citation and bibliographic coupling themes, older themes remain current. In that regard, research about the health benefits of chia seeds and their byproducts is both retrospective as well as current research since it was identified in co-citation (cluster 4) and bibliographic coupling (cluster 2) theme analysis. Yet, the functional properties of chia proteins were identified in clusters 3 and 2 of the co-citation and bibliographic coupling themes, respectively. The theme characterization of chia byproducts (cluster 2 of the co-citation) encompasses clusters 1, 2, and 3 of the bibliographic coupling. On the other hand, the plant derivative theme is retrospective, since it was identified solely on co-citation themes. The research on the health benefits of chia proteins and peptides was identified in bibliographic coupling, demonstrating its currency. Likewise, co-word overlay visualization also denotes that research on the health benefits of chia and the development of functional foods is current.

**Figure 6:**
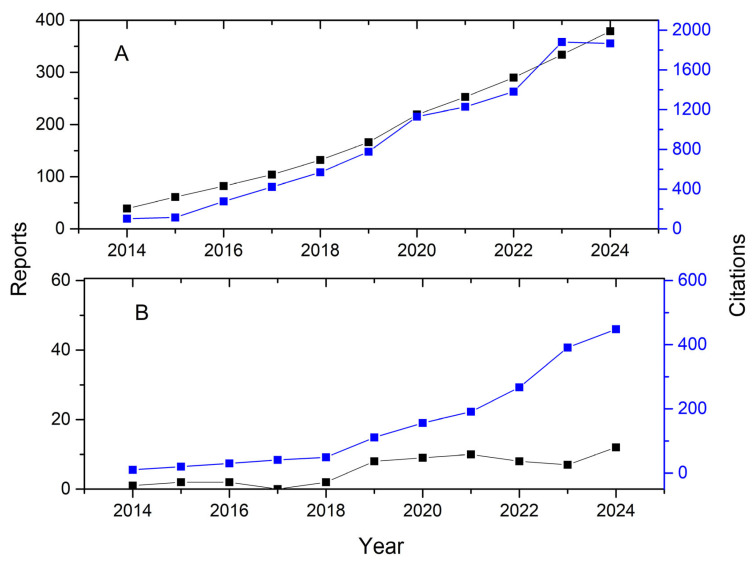
A number of reports and citations of “*Salvia hispanica*” and “protein” (**A**) and “*Salvia hispanica*” and “peptide” (**B**) from 2014 to 2024 retrieved from Scopus database.

Metrics of current research on chia proteins and peptides (Figure 6) show an increase in citations over the years for both chia proteins and peptides. Regarding the number of publications, there is a higher number for chia proteins, mainly in 2020, accounting for 53 documents. In addition, there are over 1000 citations for chia proteins, showing their current relevance. As for chia peptides, the number of publications is at or below 10 per year. Despite that, there is a growing number of citations of chia peptides from 2020, which indicates it is an emergent theme. These data corroborate the bibliometric analysis that shows the current relevance of chia proteins and chia peptides. Since current investigations are mostly directed to the functional properties of chia proteins and functional foods based on chia peptides, a general overview of these byproducts’ characteristics will be hereafter approached.

## 4. Chia Proteins

**Figure 7:**
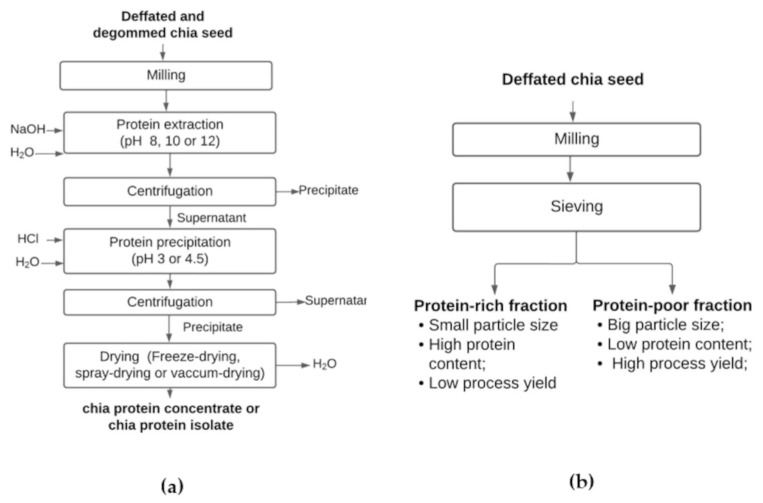
Flowchart of chia protein concentrate or chia protein isolate production by alkaline extraction followed by isoelectric precipitation (**a**) and flowchart of protein-rich fraction production by dry fractionation process (**b**).

Protein-rich fractions (PRFs), protein concentrates (CPCs), and protein isolates (CPIs) are obtained from defatted chia seed flour [35], as shown in Figure 7. Alkaline extraction followed by isoelectric precipitation (AE-IP) has been employed to obtain CPCs or CPIs from defatted flour or protein-rich fractions [4,35]. In contrast, wet or dry fractionation of chia seeds, which does not require solvent use [36], is employed to obtain chia PRFs. Dry fractionation produces PRFs with minimal protein denaturation or modification. Fractions were obtained after milling the defatted seed and separation based on the particle size and density [37]. One fraction had greater granulometry and higher fiber content. The other fraction had lower granulometry and higher protein content [24]. Increasing the mesh size reduced the extraction yield and increased protein concentration (Table 3). Fractionation at mesh 200 provided the lowest process yield (1.83%) and highest protein recovery (42.29%). A 45-mesh showed a better balance between yield and protein content [21]. Compared to the AE-IP method, dry fractionation provides lower process yields [36,37,38], which can be explained by the presence of other components in the coarse fraction that are not efficiently removed during extraction [38].

In addition, the composition of the chia seed is influenced by climate, geographical location, growing environment, humidity, and soil [39]. The protein content of chia seeds tends to increase with decreasing altitude of cultivation [40]. Moreover, chia seeds from different sources and different genotypes influenced the amino acid composition, digestibility, and thermal properties of chia proteins. In that regard, the proline content of British seed globulin was lower than the one from Mexican seed globulin [41]. Chia proteins extracted from white chia seeds had a higher content of leucine/isoleucine than chia proteins obtained from black chia seeds [42]. Chia proteins obtained from black seeds had a higher digestibility than chia proteins from white seeds, which could be related to the higher content of sulfur amino acids in the former [42]. Furthermore, seed processing to obtain chia proteins influenced the anti-nutritional factor content. Chia proteins had a lower concentration of phytic acid and a higher concentration of trypsin inhibitor regarding chia flour. Phytic acid and trypsin inhibition may impair mineral absorption and protein digestibility, respectively, and therefore nutrient absorption [41]. Regarding thermal properties, proteins extracted from two different genotypes of chia seeds showed small differences in endothermic peaks [42].

AE-IP is the most traditional method used for extracting plant proteins. After protein solubilization at an alkaline pH, the dissolved protein supernatant was acidified for isoelectric precipitation [35,41]. The process yields varied between 14.1% [36] and 19.2% [3], and the percentage of protein recovered depended on the pH used for solubilization and precipitation in the AE-IP method [3,35,36]. Protein structure changes occur during the extraction conditions (heating, alkali, or acidic medium) or drying processes, affecting their functional properties and nutritional value.

**Table 3:** Yield of the extraction process and properties of protein-rich fractions and chia protein concentrates.

Sample		Extraction Yield (%) *	Protein Content (%) *	Protein Solubility (%)	Water Absorption Capacity (g.g^−1^)	Oil Absorption Capacity (g.g^−1^)	Thermal Properties (°C)	Reference
PRF		14.1	49.7	PRF > CPC2 > CPC1	PRF > CPC1 and CPC2 (pH 5, 7, 9, 11) CPC1 > CP2 (pH 9, 11) CPC2 > CPC1 (pH 2, 3, 5)	2.3	ΔTd: 130.3–145.2	[36]
CPC1		17.1	70.9			3.2	ΔTd: 32.0–145.7	
CPC2		19.2	74.1			3.7	ΔTd: 47.3–129.8	
CPI10		11	78.2	-	-	-	Tpeak: 57 and 105	[35]
CPI12		17	75				Tpeak: 78, 94, and 112	
SDCPI		16.2	90.5	Higher at pH 8–12 Higher in NaCl 1 M	2.3	2.7	-	[24]
FDCPI		18.2	91.2		2.9	3.3		
VDCPI		17.4	90.3		2.1	3.6		
CPI10		-	-	90% (pH 7) 68% (pH 7)	4.4	7.1	-	[43]
CPI12					6	6.1		
CPI8.5		15.9	44.29 **	Higher at pH 7–11		5.24	-	[3]
CPI10		16.87	54.31 **		CPI8.5 > CPI10 > CPI12	6.52		
CPI12		19.10	59.63 **			7.55		
CPI10		-	78.2	87%	-	-	-	[44]
CPI12			77.5	33%				
PRF	mesh			60% at pH 10	-	-	-	[37]
	45	55	25.7					
	100	3.22	30.53					
	200	1.83	42.29					
PRF		~ 18	44.6	Higher at pH 10	-	-	-	[38]
Mexican CPC		-	88.32 **	-	-	-	-	[41]
British CPC			89.90 **					
Mexican albumin fraction			37.96 **					
British albumin fraction			56.75 **					
Mexican globulin fraction			44.22 **					
British globulin fraction			39.34 **					
WCPI ***		15.43%	90.00	67.30	3.00	2.20	Tpeak: 75.49 and 97	[42]
BCPI ***		15.96%	90.65	69.75	3.18	2.39	Tpeak: 75.9 and 103.0	
CPI		-	-	85.7 (pH 12) 97.1 (pH 8)	-	-	-	[5]

* PRF: protein-rich fraction; CPC: chia concentrated protein; SDCPI: spray-dried chia seed protein isolate; FDCPI: freeze-dried seed protein isolate; VDCPI: vacuum-dried seed protein isolate; AE-IPI: alkaline extraction–isoelectric precipitation; CPC1: chia concentrated protein 1; CPC2: chia concentrated obtained at pH 10; CPI8.5: chia seed protein isolate obtained at pH 8.5; CPI10: chia seed protein isolate obtained using solubilization at pH 10; CPI12 chia seed protein isolate obtained using solubilization at pH 12; % yield: g extracted powder/100 g partially defatted chia seed; % protein: g protein/100 g of extracted powder; ΔTd: denaturation range temperature; Tpeak: endothermic peak; ** calculated by the following: g of protein in chia protein/g of protein in defatted chia flour; ***: White chia protein isolate; Black chia protein isolate.

### 4.1. Amino Acid Composition and Molecular Weight

**Figure 8:**
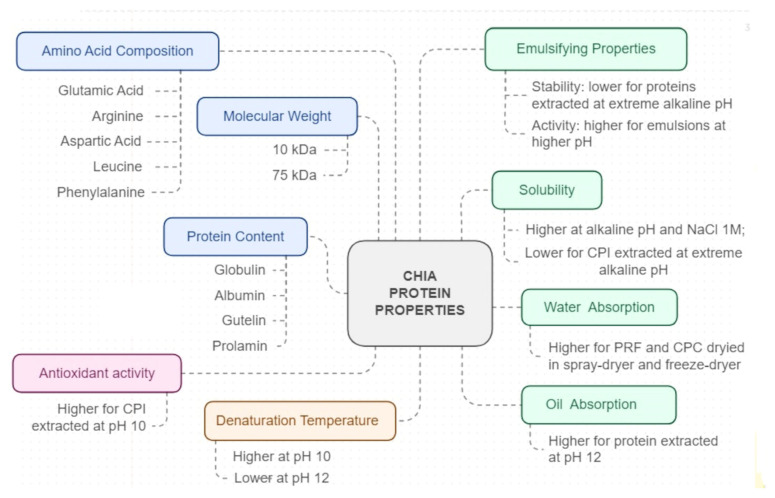
Chemical composition, techno-functional, and antioxidant properties of chia protein concentrates.

Figure 8 summarizes the overall properties of PRFs, CPCs, and CPIs obtained from chia seeds: chemical composition (protein type, amino acid composition, and molecular weight), techno-functional properties, and antioxidant activity of chia proteins [3,35,36,37,38]. Chia is a source of all essential amino acids [25] and sulfur amino acids [18,31]. PRFs and CPCs obtained from chia seeds have high glutamic acid, arginine, aspartic acid, leucine, and phenylalanine content [23,25,30,33]. The protein fractions reported in PRFs and CPCs include mainly globulin [15,35,41] and albumin [4,41,45]. While glutelin and prolamin were reported as the remaining fractions [37]. Differences in the protein fractions of protein concentrates may be due to different extraction methods and the botanical sources of chia seeds [46]. Protein isolates from two chia seed genotypes showed different variations in the leucine-to-isoleucine ratio [42].

The molecular weight of the chia protein isolate ranged from 10 kDa to 75 kDa [15,23,36]. Bands between 25 and 35 kDa are related to the prolamin fraction [1]. Bands between 20 kDa and 30 kDa have been described as globulin fractions [35,36], whereas the glutelin fraction is reported to be between 15 and 16 kDa [41]. Furthermore, the molecular weights of PRFs and CPIs differed depending on the extraction conditions. For instance, PRFs produced by dry fractionation showed a high intensity of bands between 12 and 14 kDa, whereas CPIs produced by isoelectric protein precipitation at pH 3.0, with an additional heating step at 90 °C, showed bands in the range of 20–30 kDa [36]. Changes in band intensity in electrophoresis have been denoted as the unfolding of chia protein in alkaline extraction due to its denaturation state [24].

### 4.2. Techno-Functional Properties

Co-occurrence mapping (Figure 2) evidenced that chia proteins are better investigated regarding functional properties and also named techno-functional properties. The techno-functional properties of plant proteins evaluate their applicability in food products, including their solubility, water, and oil absorption capacity, as well as their emulsifying, foaming, and gelling properties [1]. Water absorption capacity (WAC) measures the amount of water retained by one gram of protein and increases in the presence of hydrophilic components, while oil absorption capacity (OAC) measures the amount of oil absorbed by one gram of protein and increases in the presence of hydrophobic amino acids [24]. A higher OAC was obtained for CPIs extracted at a higher pH due to protein denaturation and the exposure of hydrophobic groups [3]. The increase in OAC as a consequence of protein denaturation [23,36] is caused by protein dispersion heating [36] or the protein drying process [23]. Greater surface hydrophobicity is described for CPIs with a higher OAC [23,43]. In turn, higher WAC values were observed for PRFs [36] and CPCs obtained after the spray-drying and freeze-drying of protein solutions, whereas CPCs obtained by vacuum drying showed lower WAC. Table 3 summarizes the physico-chemical properties of the protein-rich fractions and chia protein concentrates.

Solubility is affected by hydrophobic and ionic interactions, which are determined by the protein structure. At a pH close to the isoelectric point, chia proteins have lower solubility [3,38,44] As the pH increases, solubility increases [34] owing to the increased protein–water interaction [3]. Nevertheless, a very high pH induces denaturation, causing CPI aggregation and consequently lower solubility [3,44]. In that regard, CPIs extracted at pH 12 had a lower solubility than CPCs extracted at a lower pH [3,35] owing to the exposure of hydrophobic protein groups as a result of denaturation [3] Another factor affecting chia protein solubility is sodium chloride concentration. The use of 1 M NaCl increased CPI solubility, whereas the use of 2 M NaCl decreased its solubility. Increased salt concentration favors protein–salt interactions that impair protein–water interactions, consequently causing a decrease in solubility [38]. Therefore, protein solubility is a factor of major importance, as it affects its functionality [43].

Adsorption of chia protein at the air/water interface occurred more rapidly than the rearrangement of protein. The reduction in surface tension due to rearrangement was more pronounced for protein extracted at pH 10 compared to that extracted at pH 12 [44]. Furthermore, in terms of interfacial rheology, CPIs at pH 10 exhibited higher surface dilational modulus values, indicating greater intra-protein flexibility and enhanced network strength. This results in a more robust film formation, offering improved stability [44].

Moreover, emulsifying activity is influenced by pH levels. At a lower pH, protein solubility decreases, leading to reduced emulsifying activity [3,46]. Conversely, at higher pH levels, emulsifying activity increases [3,46]. Thus, PRFs derived from chia seeds demonstrated the highest emulsification ability at pH 8 and the lowest at pH 4 [38]. Increased protein solubility facilitates the rapid migration of protein to the oil–water interface, allowing them to effectively cover the droplet surface [44]. In addition, the acidic and alkaline condition of AE-IP can cause protein denaturation, which in turn impacts emulsifying properties. In this context, PRFs produced through dry fractionation exhibited better stability compared to CPCs, which showed stability and emulsifying activity of less than 20% [43].

Another important aspect of emulsions is their stability. As with emulsifying capacity, stability is also related to pH [3,46]. An increased pH decreases interfacial energy, causing a greater repulsion between adjacent droplets, which consequently increases stability [3]. Lower solubility may cause lower stability and a tendency to flocculation and cremation phenomena [43,46]. Accordingly, emulsions containing PRFs prepared at pH 9 [35] and emulsions containing CPIs extracted at an alkaline pH of 10 had greater stability [43]. Despite this, the use of extremely alkaline conditions for protein extraction resulted in CPIs with lower emulsifying stability. Emulsions containing CPIs extracted at pH 12 showed a larger particle size during storage. The extraction process and surface hydrophobicity of chia protein may account for the lower stability of emulsions [43].

### 4.3. Biological Activity

Besides functional properties, chia proteins are linked to some health-beneficial activities, including the ability to scavenge free oxygen radicals by increasing DPPH (2,2-diphenyl-1-picrylhydrazyl) and ABTS (2,2′-azino-bis(3-ethylbenzothiazoline-6-sulfonic acid) radical scavenging [3] to reduce the inflammatory process due to its ability to inhibit nitric oxide production [4] and the inhibition of the angiotensin-converting enzyme (ACE), causing a reduction in blood pressure [5].

As can be observed from bibliometric analysis (Figure 2), health benefits are more explored for chia peptides in relation to chia proteins. Proteins must be broken down into peptides and amino acids to be absorbed from the gastrointestinal tract [4] which may be the reason why there are more applications for chia peptides. Despite that, a few reports have shown the antioxidant ability of chia proteins. Notable, chia protein extracted at pH 12 exhibited lower antioxidant ability compared to chia protein extracted at pH 10, likely due to a higher degree of denaturation at elevated pH levels, resulting in amino acids lacking free-radical scavenging ability. Conversely, the chia protein isolate extracted at pH 10 demonstrated superior antioxidant capacity relative to that extracted at pH 8, attributed to its favorable amino acid profile. The chia protein isolate obtained at pH 10 had increased concentrations of tyrosine, histidine, tryptophane, and phenylalanine, which are amino acids known to be associated with antioxidant activity [3]. In addition, lower antioxidant ability was reported in chia protein concentrates regarding albumin protein fraction, which may be due to the content of phenolic compounds found in this fraction. On the other hand, chia protein concentrates showed a better ability to inhibit nitric oxide production regarding albumin fraction [4]. The chia protein isolate extracted at pH 10 had a greater ability to inhibit ACE than the chia protein isolate extracted at pH 8 due to the higher content of hydrophobic amino acids in the former [5].

### 4.4. Food Application

Studies concerning the use of chia protein in food products are scarce. Chia protein Pickering emulsions added to ice cream showed a high particle size which changed its rheological behavior. There was a low viscosity at low shear rates, and higher viscosity at higher shear rates. However, the ice cream containing the chia protein isolate had a lower hardness than an ice cream containing Pickering emulsions stabilized by a soybean protein isolate. Since the reduction in hardness is desirable regarding sensory acceptance, a chia protein isolate may be an interesting alternative to a soy protein one in the development of ice creams based on Pickering emulsions [47]. As chia proteins may have high OAC [30], they are suitable for ice cream development [48]. Proteins with high OAC can effectively bind oil, providing a stable emulsion with a desirable texture. Studying OAC provides information about protein interaction with hydrophobic molecules, helping the development of protein formulations, including plant-based and fat-replacement products. Moreover, high OAC may improve flavor retention, which consequently may improve the sensory properties [6].

## 5. Chia Peptides

Hydrolysis of chia proteins is conducted to obtain peptides [8,12,49,50], specifically CPIs [8,9], PRFs [49], or CPCs [12,50]. Chia flour [13] or defatted flour [50] are also utilized as a source of hydrolysates. Another by-product, chia expeller, is employed as a source of peptides [50,51,52]. From commercial sources, chia expeller is a subproduct from chia oil extraction, comprising 6–8% of oil [52] which is defatted before hydrolysis [50,52].

**Figure 9:**
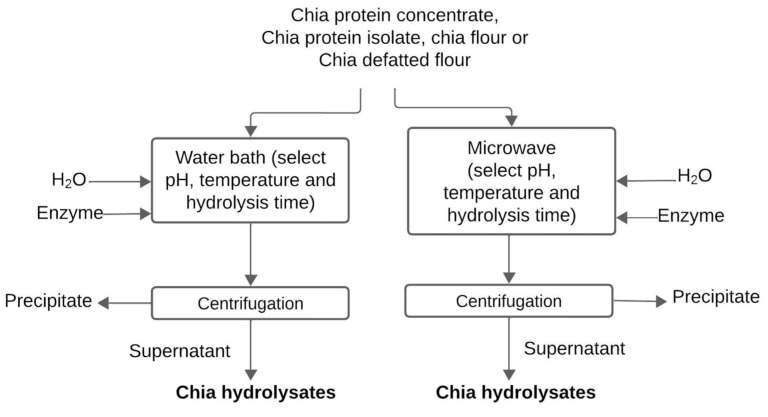
Flowchart of chia peptide production by water bath or microwave using enzymes at different pH, temperature, and hydrolysis time.

Various enzymes may be employed, including alcalase [9,52,53] flavourzyme [9,52] pepsin [9,12], trypsin [9], and papain [42,49]. Alcalase [8,9,52,54] and flavourzyme [8,52] are the most frequently utilized enzymes for hydrolysis. Alcalase is one of the most used enzymes [8,9] due to its high capacity to hydrolyze the peptide bonds of hydrophobic residues, rendering it suitable for chia protein hydrolysis [52]. Conversely, flavourzyme is an enzyme with a broader specificity [11]. Typically, alcalase and flavourzyme are employed in an enzyme/substrate ratio of 0.3 U g^−1^ and 50 U g^−1^, respectively [52]. Additionally, varying temperatures, pH, and hydrolysis times are employed, as illustrated in Figure 9. The hydrolysis degree increases with longer hydrolysis time and higher heating temperature [51,55]. Nevertheless, a hydrolysis degree of at least 50% was obtained in the absence of heating [51]. Sequential hydrolysis combining alcalase and flavourzyme is also used to increase the hydrolysis degree in comparison with isolated enzymes [11,13,49,56,57]. Predominantly, chia peptides are obtained in a water bath, while microwave-associated sequential hydrolysis has been proposed as an effective method for optimizing peptides’ biological activity in comparison to hydrolysis in a water bath [57]. Table 4 summarizes the enzymes employed and hydrolysis conditions for peptide production, while Figure 9 illustrates the overall process for peptide production. Generally, hydrolysis times range from 60 min [49] to 4 h [56] (Table 4), while pH is dependent on enzyme type [9,14,50,52]. Temperatures employed range between 37 °C [9,14] and 50 °C [49,52,56]. Therefore, various enzymes, pH, and temperatures may be employed and should be predetermined.

Regarding amino acid composition, chia hydrolysates exhibited a high content of hydrophobic amino acids, which accounts for their antioxidant activity, namely alanine, valine, methionine, isoleucine, leucine, phenylalanine, proline, and tyrosine. Aromatic amino acids such as histidine and tryptophan are also found in chia protein hydrolysates [54]. Concerning molecular weight, hydrolysates demonstrate a molecular weight of 4.2 kDa, while isolated chia protein weighs 15 kDa, which is an expected effect since hydrolysis reduces molecular weight [8,57]. Peptides containing between 2 and 20 amino acids are associated with biological activity [8], and therefore, molecular weight is also a crucial factor for the antioxidant activity of peptides [57].

### 5.1. Functional Properties

Although the co-word network (Figure 2) has not shown a link between functional properties and peptides, some reports have demonstrated the potential techno-functional properties of peptides [8,12]. The co-word occurrence map was created from keywords defined by the author, and the term techno-functional hydrolysate was defined as a keyword by reports showing the functional properties of chia peptides [8,12], which justifies the absence of a link between peptide and functional properties in the co-word occurrence network. This fact evidences that the full reading of reports selected for bibliometrics is fundamental to identifying information not displayed by bibliometrics solely.

**Figure 10:**
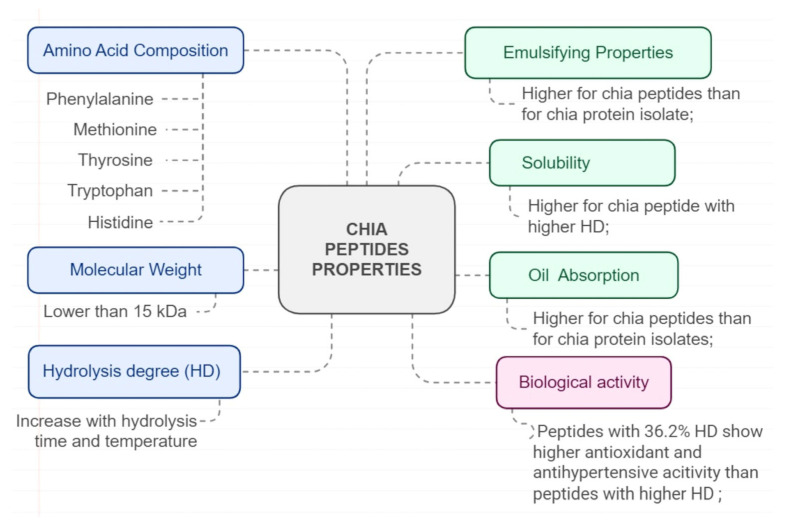
Chemical composition, techno-functional, and biological properties of chia protein peptides.

Chia protein isolates and chia peptides exhibited similar water absorption capacity, while oil absorption capacity was higher for peptides than for chia protein isolates [8]. Chia isolated protein and chia hydrolysates had a trimodal and unimodal particle distribution [5], which may accomplish a greater stabilizing effect and emulsifying activity of peptides regarding the protein isolated [8]. Figure 10 summarizes the main properties of chia peptides.

Enzymatic hydrolysis produces an amphipathic protein derivative [50]. Hydrophobic groups may contribute to the higher oil retention capacity and consequently to emulsifying properties, while hydrophilic groups may enhance peptide solubility [44]. Consequently, chia hydrolysates exhibited greater surface hydrophobicity due to the exposure of hydrophobic groups [12]. Thus, peptides demonstrated increased emulsifying activity due to the reduction in interfacial tension [55], resulting in an emulsion with a smaller droplet size and consequent greater stability [8,12] The enhanced stability of emulsions based on chia hydrolysates occurs at higher pHs due to their increased surface hydrophobicity and their greater availability to act on the emulsion interface [12]. Table 4 summarizes the main properties of chia peptides.

Furthermore, hydrolysates may exhibit a broader solubility than chia proteins [5]. Peptides extracted at longer hydrolysis times demonstrated higher solubility due to an increased degree of hydrolysis. Peptides obtained at a hydrolysis time exceeding 60 min had a more than 45% hydrolysis degree and an average solubility of 70 µg/g of protein. Similarly, a combination of sequential enzymatic extraction with microwave treatment increased peptide solubility at pH 3 and 5, which is proximal to the isoelectric point of chia proteins and generally the pH at which the protein is least soluble [8,12].

**Table 4:** Enzymes and extraction conditions employed to obtain chia peptides.

Chia Byproduct	Extraction Conditions		Hydrolysis Degree (HD)	Reference
	Enzyme	Time and Temperature		
CPI *	Alcalase (pH 8)	3 h at 50 °C	Highest HD (~35%) with alcalase	[9]
	Pepsin (pH 8)	3 h at 37 °C		
	Trypsin (pH 8)	14 h at 37 °C		
	α-chymotrypsin (pH 8)	14 h at 37 °C		
PRF *	Alcalase (pH 7) or flavourzyme (pH 8)	Until 150 min at 50 °C	Sequential hydrolysis improved HD (~40%)	[41,49]
	Sequential hydrolysis: alcalase (pH 7) and flavourzyme (pH 8)	60 min (alcalase) and 150 min (flavourzyme)		
Chia expeller	Papain (pH 7)	3 h at 45 °C	~15% after 120 min	[50]
Chia expeller	Alcalase (pH 7)	Until 240 min at 50 °C	HD greater than 50% for sequential extraction	[52]
	Flavourzyme (pH 8)	Until 240 min at 50 °C		
	Sequential hydrolysis: alcalase (pH 7) and flavourzyme (pH 8)	90 min (alcalase) and 0–240 min (flavourzyme)		
CPC PRF Chia flour	Alcalase (pH 7)	Until 240 min at 50 °C	HD over 20% for sequential hydrolysis Higher HD to peptides obtained from CPC	[56]
	Flavourzyme (pH 8)	Until 240 min at 50 °C		
	Sequential hydrolysis: alcalase and flavourzyme	60 min (alcalase) and 180 min (flavourzyme)		
CPI	Sequential hydrolysis: pepsin (pH 2) and pancreatin (pH 7.5)	45 min (pepsin) and 45 min (pancreatin) at 37 °C	HD over 38%	[14]
Chia flour	Sequential hydrolysis or isolated: alcalase or flavourzyme (pH 8)	Maximum of 90 min until reaching 95 °C	HD greater than 40% to sequential hydrolysis	[57]
CPC	Alcalase (pH 8)	15 min at 50 °C	-	[58]
	Pepsin (pH 2)			
	Pancreatin (pH 7.5)			
CPC	Sequential hydrolysis: pepsin (pH 2) and pancreatin (pH 7.5)	Reaction stopped after 20 min at 80 °C	HD of 51.1% to sequential hydrolysis	[12]
CPI	Alcalase (pH 8)	Until 6 h at 50 °C	Highest HD in the longest hydrolysis times	[53]
	Bromelain (pH 7)			
	Papain (pH 7)			
CPI	Thermolysis (pH 2)	Until 5 h; reaction stopped after 5 min at 95 °C	Highest HD to 0.02 NTris-HCl + 10 mM CaCl_2_ buffers at 2 h of hydrolysis	[5]
PRF	Sequential hydrolysis: pepsin (pH 2) and pancreatin (pH 7.5)	45 min (pepsin) and 45 min (pancreatin) at 37 °C	HD >30%	[59]
PRF *	Sequential hydrolysis: pepsin (pH 2) and pancreatin (pH 7.5)	45 min (pepsin) and 45 min (pancreatin) at 37 °C	-	[60]

* PRF: protein-rich fraction, CPC: chia concentrated protein, CPI: chia protein isolate.

### 5.2. Biological Applications

Chia peptides have also been associated with antioxidant [52,53], anticoagulant [52], anticancer [61], antidiabetic [5,58], and anti-inflammatory activities [14]. Generally, the increment in hydrolysis time increased the hydrolysis degree and improved antioxidant activity and ACE inhibition. As expected, longer hydrolysis time and heating temperature lead to a greater degree of hydrolysis [51,55]. However, the highest hydrolysis degree may diminish certain biological properties [11,55]. Extensive hydrolysis resulted in peptides with reduced antioxidant ability or ACE inhibition [11], possibly due to the oxidation of histidine or tryptophan. These amino acids are related to ACE inhibitory activity [62] and to antioxidant action [63]. Hydrolysis with alcalase (for 60 min) and flavourzyme (for 150 min) favored the highest degree of hydrolysis (43.8%). The ability to scavenge free oxygen species is also improved by the hydrolysis process since non-hydrolyzed forms showed a lower antioxidant ability [52,53].

Peptides obtained through sequential hydrolysis demonstrated higher anti-hypertensive, anticoagulant, and antioxidant ability compared to peptides obtained by isolated enzyme hydrolysis [52]. Additionally, anticancer activity against breast cancer, liver cancer, and colorectal cancer cell lines was demonstrated mainly for peptides obtained through sequential hydrolysis with pancreatin and pepsin [59]. Although sequential extraction may be preferable for obtaining hydrolysates with greater ability to interact with endogenous molecules and receptors [52], hydrolysis with isolated enzymes also yields peptides with beneficial health effects. In this regard, alcalase-obtained peptides and bromelain-obtained peptides exhibited a superior ability to inhibit dipeptidyl peptidase IV, whose inhibition is associated with an antidiabetic effect [53].

The presence of hydrophobic amino acids has been reported to promote the beneficial biological activities of peptides [9,53,59,64]. Indeed, pepsin protein hydrolysate demonstrated superior ACE inhibitory activity compared to trypsin hydrolysate, chymotrypsin hydrolysate, and alcalase hydrolysate, likely due to its hydrophobic amino acid content [9]. Beyond the amino acid composition, another important feature is the molecular weight of peptides. Usually, molecular weights ranging from 1 to 3 KDa are related to better biological effects [14]. Peptides from sequential hydrolysis with lower molecular weights showed a reduction in mice ear edema in addition to lower delayed hypersensitivity induced by Dinitrofluorobenzene, denoting its superior anti-inflammatory activity over fractions with higher molecular weight [14]. Similarly, peptides from sequential hydrolysis bearing 1 kDa had a higher cytotoxicity on cancer cell lines, which shows its better anticancer ability [59]. Recent reports based on in silico modeling focus on identifying the composition of amino acids, the peptide’s molecular weight, and the binding energy to target receptors as strategies for predicting their effect in vivo [64].

### 5.3. Food Applications

Chia peptides were added to bread due to the absence of gluten in chia proteins. Replacing gluten can change the viscoelasticity and sensorial characteristics of bread, thus affecting the food quality and its acceptance by consumers [65]. Nevertheless, panelists did not observe a difference between peptide-enriched bread and bread without peptides [51]. The fortification of bread with a higher peptide content increased specific volume and caused no difference in hardness regarding bread devoid of peptides. Lower hardness and higher specific volume may be related to greater sensory acceptance. Accordingly, bread with peptides received a sensory score above 80, indicating that it shows very good acceptability [55]. In addition, the partial replacement of wheat flour by chia peptides did not cause a decrease in bread color, as would be expected for chia derivatives [66]. The lower lysine content in peptides may contribute to lower darkening. Then, in terms of color, hydrolysates may have an advantage over other chia derivatives, as color is an important sensory acceptance factor [67]. Despite the interesting sensory features, the addition of peptides extracted in 150 min of hydrolysis time into bread provided lower ACE inhibition ability than bread containing peptides obtained from 90 or 120 hydrolysis time. The antioxidant activity was neither improved by peptide addition to bread. Bread cooking may have caused the oxidation of histidine and tryptophan and therefore reduced the antioxidant ability [55]. In addition to extraction methods, operations employed in food processing can also affect peptide features.

Chia hydrolysates were also added to carrot creams. Antioxidant activity in cream with hydrolysates was higher than in control carrot cream. However, peptide content and the extraction conditions (time and temperature) did not affect the antioxidant activity and ACE inhibition of carrot creams. Furthermore, hydrolysis reduces the interaction between amino acids and water, consequently impacting rheology. A chia peptide content of 5 mg/g led to carrot cream with a higher consistency index and lower flow index, while a content of 2.5 mg/g of chia peptide did not cause flow changes in the food. Furthermore, carrot cream containing 5 mg/g of peptides presented a similar sensory score to that of the carrot cream devoid of peptides, while the carrot cream containing 2.5 mg/g of peptides presented a lower score. These data show the possibility of using higher concentrations of peptides without affecting sensorial acceptance [55].

## 6. Future Perspectives

Despite the challenges in achieving chia protein with suitable stability, several reports have shown the potentialities of chia proteins and peptides [62]. Aspects related to extraction method optimization as well as scaling up need to be better investigated to produce chia protein derivatives with technical and economic feasibility, as well as desirable biological and technological features. Recently, in vitro digestibility assays have been applied to understand the protein and peptide absorption process throughout the gastrointestinal tract and the effects of digestion processes on bioactive compounds [4]. Bioinformatics methods has also emergent to elucidate biological activity [68]. More in-depth investigations of chia protein digestibility and bioactivity are expected to enable the development of nutraceuticals. Furthermore, chia protein derivatives may be employed as a natural source preservative of foods, due to their ability to inhibit the growing of gram-positive bacteria *Salmonella* [69] *and Listeria monocytogenes* [70], and therefore can improve the shelf life of meats [69] and dairy products [70]. These protein derivatives are also interesting for preventing foodborne infections caused by these bacteria [69,70].

Besides the food industry, chia protein shows potential in the pharmaceuticals industry due to its inhibition of the angiotensin-converting enzyme, a target of antihypertensive drugs [3], as well as its ability to inhibit dipeptidyl-peptidase-4, a target of antidiabetic drugs [5]. Chia proteins could also be employed in the development of anti-aging cosmetics due to their antioxidant ability [3,5] and due to collagenase and elastase inhibition [71]. Furthermore, due to the inhibition of tyrosinase activity, chia protein derivatives could be employed for hyperpigmentation-reduction cosmetics [71].

The Co-word map showed that antioxidant ability is one of the most researched biological features of chia proteins and peptides and that it can be further investigated in the treatment of diseases such as cancer and cardiometabolic diseases. A low-strength link between health benefits and functional properties shows the feasibility of obtaining chia protein derivatives combining both health benefits and techno-functional features. The few reports on chia peptides’ techno-functional properties were obtained from the chia protein isolated, using alcalase and/or flavourzyme [8,54]. In turn, the health benefits of chia protein were reported from the isolated chia protein [3], chia protein concentrates, and protein fractions [4]. Therefore, there is a need for further research on the functional properties of peptides obtained from different protein sources, as well as from different hydrolysis processes. Furthermore, proteins whose antioxidant, anti-inflammatory, and angiotensin-converting enzyme inhibition ability was shown were extracted through alkaline extraction–isoelectric precipitation. Novel eco-friendly methods can be employed to obtain derivatives with lower environmental impacts and allowing extraction under less drastic conditions.

## 7. Conclusions

Bibliometrics is a valuable scientometric tool to highlight the current research status. In that regard, chia protein and peptide research was identified as a current theme. Mainly, the investigation of peptide properties is an emerging theme that should progress in the near future. In addition, the analysis of the co-word occurrence map together with the overall review of chia protein and peptides shows that antioxidant activity is one of the most studied properties. The concomitance between health benefits and functional properties was poorly shown, evidencing the need for further investigations on chia protein/peptides with functional properties and health benefits. Despite the potential of bibliometrics, its limitations are related to the risk of bias of the selected reports as well as the use of citations as a metric for bibliometrics, which may also limit research tracking. The highlighted findings are limited to the reports selected for this review, which may require a new future review to update new articles published. Systematic reviews would be useful to indicate the evidence quality of reports on chia protein derivatives.

The use of chia protein derivatives as emulsifying/co-emulsifying agents represents a potential application in the food industry, and the appropriate process conditions must consider amino acid profile, solubility, surface hydrophobicity, denaturation, emulsifying properties. Extraction of chia protein isolates and chia protein concentrates under higher pH increased surface hydrophobicity and reduced solubility, which may impair chia protein’s ability to stabilize emulsions. In turn, for chia peptides, a greater hydrolysis degree may improve its emulsifying activity but may decrease its biological activity.

Baked products are the main food category for chia peptides, due to the gluten-free profile of chia seeds. On the other hand, the chia protein isolate was applied only to ice cream due to its emulsifying ability. However, other food categories could also be used, due to the improvement in techno-functional properties or health benefits. Furthermore, chia protein enrichment in foods would be an interesting daily source of proteins in vegan people. Besides the food industry, chia proteins show promising application in the pharmaceutical and cosmetic industry. Therefore, extraction conditions must be optimized and selected depending on the desired technological or biological characteristics. The research trend for peptides can guide researchers in directing funding to this area and to a better investigation of in silico methods and in vitro digestibility, as well as in exploring the application of chia protein derivatives for several oxidate stress-related disorders.

## Data Availability

No new data were created or analyzed in this study. Data sharing is not applicable to this article.
